# Diversity of extracellular vesicles derived from calli, cell culture and apoplastic fluid of tobacco

**DOI:** 10.1038/s41598-024-81940-8

**Published:** 2024-12-03

**Authors:** Michaela Kocholatá, Jan Malý, Sylvie Kříženecká, Olga Janoušková

**Affiliations:** 1 Centre for Nanomaterials and Biotechnologies, Faculty of Science, Jan Evangelista Purkyně University in Ústí Nad Labem, Ústí Nad Labem, Czech Republic; 2 Department of Environmental Chemistry and Technology, Faculty of Environment, Jan Evangelista Purkyně University in Ústí Nad Labem, Ústí Nad Labem, Czech Republic

**Keywords:** Biotechnology, Cell biology, Plant sciences

## Abstract

In recent years, there has been a growing interest in plant extracellular vesicles (pEVs) due to their immense potential for medical applications, particularly as carriers for drug delivery. To use the benefits of pEVs in the future, it is necessary to identify methods that facilitate their production in sufficient quantities while maintaining high quality. In this study, a comparative analysis of yields of tobacco pEV derived from apoplastic fluid, sterile calli, and suspension cultures, was performed to identify the most suitable plant material for vesicle isolation. Subsequent experiments focused on assessing the efficiency of small interfering RNA (siRNA) loading into callus-derived vesicles, employing various methods such as sonication, incubation, incubation supplemented with saponin, lipofection, and electroporation. Differences in loading efficiency among vesicles derived from apoplastic fluid, calli, and suspension cultures were observed. Moreover, our investigation extended to the presence of tobacco secondary metabolites, specifically anabasine and nicotine, within vesicles originating from three distinct tobacco sources. The outcomes of our study highlight variations not only in vesicle yields based on their source but also in their loadability and the presence of nicotine and anabasine. These findings contribute valuable insights into optimizing the production and application of pEVs for future medicinal purposes.

## Introduction

In the field of plant biology, recent discoveries span from entire plants to their cells and subcellular structures. Within plant cells, sophisticated molecular interactions regulate growth, development, and responses to environmental stimuli. Recent studies have revealed a new dimension of cell communication: plant extracellular vesicles (pEVs)^1,2^. These small membrane vesicles, acting like nature’s delivery service, facilitate information exchange between plant cells, similar to those in animals and microbes^3–6^.

Plants are known for their adaptive signaling mechanisms in response to environmental stimuli. The discovery of pEVs has added complexity to our understanding of plant environmental coordination^7^. Plant extracellular vesicles have gained attention for their role in intercellular communication and physiological processes^6,8–11^. Phospholipid membrane of pEVs encapsulate a diverse cargo, including proteins, lipids, nucleic acids (mainly small RNAs), and secondary metabolites. The precise composition of pEVs can exhibit variation contingent on factors such as environmental conditions, the source of pEVs or the state of the source organism^5,12–14^.

The significance of plant EVs lies in their role as messengers mediating intercellular signals. They facilitate responses to environmental stressors, pathogens, and developmental cues, carrying bioactive molecules that influence immune responses, growth regulation, and stress tolerance. Plant EVs are crucial in transferring defense-related molecules, enabling neighboring cells to prepare for various abiotic and biotic stresses^7,15,16^. In addition to their fundamental roles in plant biology, pEVs have opened door to innovative biotechnological applications. Researchers are actively exploring the potential of these vesicles for the targeted delivery of cargo, ranging from genetic material to various bioactive compounds^17,18^. In biopharmaceuticals, traditional drug delivery systems often face challenges like stability, immunogenicity, and controlled release. Plant EVs, capable of crossing biological barriers and targeting specific cells, offer a promising alternative. They can encapsulate a wide range of therapeutic agents, enhancing drug efficacy and reducing side effects^19–24^. It was already shown, pEVs are able to deliver endo- and exogenous agents into mammalian cells in vitro and *in vivo*^12,25,26^. PEVs from edible plants are particularly promising due to their abundance, biocompatibility, and biodegradability. They hold potential as a cell-free therapeutic approach for various diseases. Additionally, secondary metabolites within pEVs, derived from medicinal herbs, fruits, or vegetables, provide added value with properties such as anti-inflammatory, anti-cancer, and antioxidant effects^12,27–29^.

To use their potential, there arises a critical need to produce pEVs in sufficient quantities. PEVs are typically isolated from a variety of sources, with fruit and vegetables, particularly their juices, being common starting points for extraction. However, they can also be obtained from apoplastic fluid and plant explant cultures, as previously demonstrated^30–32^. Moreover, in certain instances, researchers have successfully isolated plant extracellular vesicles from plant mixates^9^. This diversity in isolation methods and sources underscores the versatility and potential applications of these vesicles across the realms of plant biology, medicine etc.

In this research article, comparison of yields of plant extracellular vesicles derived from various tobacco sources, which include callus cultures, BY-2 suspension cultures, and apoplastic fluid, using ultracentrifugation as the primary isolation method was conducted. The BY-2 cell line, derived from the seedlings of the tobacco cultivar Bright Yellow-2, is a popular plant model that has been extensively used to study various aspects of plant physiology^33,34^. Our study highlights the difference in the yields of plant extracellular vesicles and their respective small interfering RNA (siRNA) loading capacities across these sources using different loading methods in different variations. Additionally, the presence of two tobacco secondary metabolites, nicotine and anabasine was investigated. The primary objective of this investigation is to identify the most suitable source of plant extracellular vesicles, considering their yields, the presence of secondary metabolites—nicotine and anabasine, and their siRNA loading capabilities. This selection process aims to pave the way for their optimal utilization in future applications.

## Materials and methods

### Plant material

To initiate the formation of callus of *Nicotiana tabacum*, seeds (purchased at SemenaOnline; Jeneč; Czech Republic) were surface-sterilized and placed on a Murashige-Skoog (MS) medium previously described for tobacco callus induction with some modifications^35^. This medium was enriched with saccharose (30 g/l), plant agar (8 g/l; Duchefa Biochemie, B. V; Haarlem; Netherlands), 1-Naphthaleneacetic acid (NAA; 1.2 mg/l; Duchefa Biochemie, B. V; Haarlem; Netherlands), and 6-Benzylaminopurine (BAP; 1.2 mg/l; Duchefa Biochemie, B. V; Haarlem; Netherlands). The pH of the medium was adjusted to a range of 5.6–5.8, and it was subjected to sterilization in an autoclave at 121 °C for a duration of 30 min. Cultivation of callus cultures took place within a controlled environment in a grow box (Garden High Pro Probox Propagator XL; Grow technologies; Prague; Czech Republic), maintaining a temperature of 25 °C under illumination with 16/8 photoperiod. The formation of callus typically became evident within a span of 3 to 4 weeks, after which the callus was routinely transferred to fresh medium every 6 weeks.

The BY-2 suspension culture of *Nicotiana tabacum* was generously provided by the Institute of Experimental Botany of the Czech Academy of Science. These BY-2 cultures were cultivated under illumination with 16/8 photoperiod and in the absence of light, maintaining a constant temperature of 26 °C on a shaker set at 105 RPM. To ensure their continued growth and vitality, cells were regularly transferred into fresh growth medium on a weekly basis. The growth medium itself was prepared, using Murashige-Skoog basal salt mixture at a concentration of 4.3 g/l, supplemented with KH_2_PO_4_ (0.2 g/l), saccharose (30 g/l), inositol (100 mg/l), thiamine (1 mg/l), and 2,4-dichlorophenoxyacetic acid (2,4-D; 0.2 mg/l). Carefully maintaining the pH of the medium within the range of 5.6 to 5.8, the entire mixture was subsequently sterilized in an autoclave at 121 °C for a duration of 30 min. All chemicals for media preparation mentioned above were purchased at Duchefa Biochemie, B. V; Haarlem; Netherlands.

About five *Nicotiana tabacum* seeds were sown in plastic pots measuring 7 × 7 cm, filled with gardening soil enriched with hummus. Plants were cultivated within a plant growth box set at a constant temperature of 25 °C (± °1C). The grow box was equipped with full spectrum LED lighting and operated on a photoperiod of 16 h of light followed by 8 h of darkness. To sustain their growth, the plants received watering every third day. After 28 days from planting, they were harvested, at size about 10 to 15 cm which is the optimal size for the isolation of apoplastic fluid.

### PEVs isolation

#### Collecting plant material

The process of ultracentrifugation was chosen as a method for pEVs isolation. The preparation of plant material for pEVs isolation varies according to the source material. As a universal parameter for isolating pEVs from all three types of plant material, the weight was standardized by using 10 g of each material, which was then subjected to the following analysis.

Exactly 10 g of tobacco callus, six weeks after passage, was weighed and placed into a Falcon tube with 10 ml of 1X PBS buffer. Due to the friable nature of tobacco callus, the callus could be divided into small pieces by shaking the tube for one minute. Subsequently, large particles were removed using plastic kitchen sieve (with the mesh size 1 × 1 mm) and the suspension was transferred to a centrifugation tube and subjected to centrifugation.

Similarly, 10 g of tobacco suspension culture was collected and prepared for the subsequent centrifugation steps. BY-2 suspension cultures were collected 7 days after the transfer to fresh media.

Finally, 10 g of young tobacco plants (typically 3–6 flowers) were harvested and the apoplastic fluid isolation protocol by Rutter et al. was followed^30^. Briefly, tobacco plants were carefully removed from a soil, cleaned by distilled water and placed into a metal French press filled half way with 1X PBS. French press with tobacco plants and buffer was placed into a glass exsicator and a vacuum (300–400 mbar) was applied for one minute and 20 s, facilitating the entry of the surrounding buffer into the plant’s apoplast by opening its pores. Plants were transferred into a tool made of 50 ml plastic tube with inserted 30 ml syringe, and centrifuged at 700 × g for 25 min (Beckman Coulter’s OPTIMA XPN 90 ultracentrifuge with rotor 70-Ti; Indianapolis; United States), resulting in the release of apoplastic fluid from the plants through the syringe into the 50 ml plastic tube. Collected apoplastic fluid was subjected to centrifugation steps.

#### Ultracentrifugation

The samples were initially centrifuged at 2000 × g for 20 min at a temperature of 4 °C. The supernatant obtained was collected and underwent a subsequent centrifugation at 10,000 × g for 30 min. This step was carried out using Beckman Coulter’s Avanti JXN-26 high-speed centrifuge with rotor JA-25.50 (Indianapolis; United States), and it was repeated twice to enhance sample purity. The supernatant from these rounds was collected and subjected to another centrifugation at 100,000 × g for 1 h at 4 °C. This step, performed using Beckman Coulter’s OPTIMA XPN 90 ultracentrifuge with rotor 70-Ti (Indianapolis; United States), was also repeated twice^36^. The resulting pellet was resuspended in 500 μl of 1X PBS and stored at -20 °C for subsequent analysis.

In response to difficulties encountered in resuspending the pellet, the samples were centrifuged at 5,000 × g for 5 min to eliminate large clusters of the pellet remaining in the samples.

### Nanoparticle tracking analysis

Particles within the samples were analyzed using NanoSight NS3000 (Malvern Paralytical; Malvern, United Kingdom), and videos were recorded and processed using the NTA software. Roughly 300–400 μl of each sample was introduced into the flow-cell top-plate chamber. A laser beam with a wavelength (λ) of 562 nm illuminated the chamber from below, causing the particles in the solution to scatter light. Each sample underwent three separate analyses, each lasting 30 s, while the ambient temperature remained below 25 °C. The results were evaluated with the assistance of the Malvern software.

### Loading of plant extracellular vesicles

siRNA (MISSION® siRNA Fluorescent Universal Negative Control #1, Cyanine 5; Sigma-Aldrich; Saint Louis; United States) with a fluorescent label (cy5) was selected for loading into pEVs. In each variation, the initial concentration of siRNA used for loading was 11.07 µg/ml.

In our initial set of experiments, the loading of siRNA into callus-derived pEVs, utilizing different approaches of sonication, incubation, electroporation, and lipofection was examined.

Sonication was performed using a probe sonicator (Bandelin Sonopuls mini20 homogenizer; Berlin; Germany). Sonication samples were prepared by combining 1 × 10^9^ of pEVs, 11.07 µg/ml of labeled siRNA, and samples were adjusted to a volume of 100 µl using 1X PBS. Four variations of sonication were conducted: a) 20% amplitude, four cycles (1 cycle includes sonication 30 s on, 30 s off for three minutes followed by 2 min of cooling); b) 20% amplitude, six cycles; c) 10% amplitude, four cycles; and d) 10% amplitude, six cycles^24,37^. Following sonication, the samples were subjected to a 30-min incubation at 37 °C to facilitate the reconnection of disrupted membranes. After incubation period, the removal of free siRNA was achieved through ultracentrifugation steps at 100,000 × g for one hour, 4 °C. The supernatant was discarded, and the resulting pellet was resuspended in 1X PBS, followed by a repetition of the ultracentrifugation step. Upon completion of the centrifugation, the pellet was resuspended in 50 µl of 1X PBS, and the samples were immediately utilized for NTA analysis and fluorescence measurement.

Loading via incubation, based on a procedure by Didiot et al.^38^, was conducted in two different variations, each involving four different incubation durations, before the removal of free siRNA. The first variation involved straightforward incubation without the addition of facilitating substances. Samples were prepared by mixing 1 × 10^9^ pEVs with 11.07 µg/ml of siRNA, adjusting the volume to 100 µl using 1X PBS. Samples were incubated at 37 °C for four different time durations, including 1 h, 4 h, 6 h, and 24 h. Following the incubation, the samples were cleaned of free siRNA as described above. In the second variation of incubation, saponin was added as it has been previously used for loading mammalian extracellular vesicles, demonstrating its ability to create membrane pores within EVs by interacting with membrane cholesterol and facilitating cargo loading^39,40^. The samples were prepared identically, with the addition of saponin to achieve a final concentration of 0.1 mg/ml. Saponin-supplemented samples were incubated at 37 °C for four different time periods, including 1 h, 4 h, 6 h, and 24 h. The samples underwent the removal of free siRNA as previously described.

The electroporation was performed using Nucleofector™ 2b (Lonza; Allendale, United States), choosing a gentle program designed for HeLa cells with high viability. Samples were prepared by mixing 1 × 10^9^ pEVs, 11.07 µg/ml of labeled siRNA, and adjusting the sample volume to 100 µl. After the electroporation, the samples were subjected to incubation at 37 °C for four different time intervals: 1 h, 4 h, 6 h, and 24 h to recover the membranes. Subsequently, the samples were cleansed of free siRNA using ultracentrifugation as described earlier.

For lipofection, the ultracentrifugation pellets containing 1 × 10^9^ pEVs were used. The transfection solution was prepared by combining two mixtures in a 1:1 ratio: a) a mixture of Lipofectamine™ RNAiMAX Transfection Reagent (Invitrogen; Carlsbad, United States) and Opti-MEM (Thermo Fisher Scientific; Waltham; United States) in a 1:17 ratio; b) 11.07 µg/ml of siRNA and Opti-MEM (Thermo Fisher Scientific; Waltham; United States) in a 1:6 ratio. The final solution was incubated at room temperature for 5 min. Subsequently, 100 µl of the mixture was added to each pEVs pellet. Complete samples were then incubated at 37 °C for four different time intervals: 1 h, 4 h, 6 h, and 24 h. Following the incubation, the samples were purified of free siRNA using ultracentrifugation as described above.

Following the outcomes of loading experiments conducted on extracellular vesicles derived from callus, we opted for two loading techniques to assess the loading efficiency of pEVs obtained from tobacco callus, suspension culture, and apoplastic fluid.

The first of the selected methods was sonication, following the same sample preparation procedure mentioned earlier. The sonication parameters were as follows: an amplitude of 20%, with four cycles (1 cycle includes sonication 30 s on, 30 s off for three minutes followed by 2 min of cooling). After sonication, the samples were subjected to incubation at 37 °C for 30 min. After this incubation period, the removal of free siRNA was accomplished through a two series of ultracentrifugation steps at 100,000 × g for one hour at 4 °C. The supernatant was then discarded, and the resulting pellet was reconstituted in 1X PBS. The centrifugation pellet was resuspended in 50 µl of 1X PBS, and the samples were ready for Nanoparticle Tracking Analysis (NTA) and fluorescence measurements.

The second selected method was incubation. Samples were prepared by mixing 1 × 10^9^ pEVs, 11.07 µg/ml of labeled siRNA, and 1X PBS to reach a final volume of 100 µl. These samples were incubated for three different time periods: 1 h, 6 h, and 24 h, all at 37 °C. After each respective incubation period, the removal of free siRNA was achieved through a two series of ultracentrifugation steps at 100,000 × g for one hour at 4 °C. Subsequently, the supernatant was discarded, and the resulting pellet was reconstituted in 50 µl of 1X PBS and it was used for further analysis.

### Detection of loaded cy5-siRNA

For detection of the presence of labeled siRNA in plant vesicles after loading, a fluorescence microplate reader (GloMax Discover Microplate Reader; Promega; Walldorf; Germany) was used. The volume of 50 µl of each sample of pEVs washed out of free siRNA were transferred into microplate and the fluorescence intensity was measured (excitation = 627 nm, emission = 660–720 nm). Using the same procedure, the calibration curve of siRNA was prepared to allow the calculation of the concentration of pEVs-loaded siRNA.

### LC–MS/MS analysis

LC–MS/MS analysis was performed to verify the presence of nicotine and anabasine within pEVs derived from three different sources of tobacco. Exactly 1 × 10^10^ of pEVs were taken from isolated samples from callus, suspension cultures and apoplastic fluid in triplicates. The samples were diluted several times. Separation was carried out using an Agilent 1290 Infinity II UHPLC system (Agilent Technologies; Santa Clara; United States). Chromatographic separation was achieved using a Luna Omega PS C18 analytical column 2.1 × 100 mm, 3 µm particle size from Phenomenex (Torrance; California; USA) at a flow rate of 0.4 ml/min. The mobile phases consisted of (A) H_2_O with 0.5 mM NH_4_F, pH 10 and (B) acetonitrile. The gradient was 95% A at 0 min, 70% A at 7.0% A at 9 min to 12 min, 95% A at 12.1 min. The post time was 4.9 min with 95% A and the stop time 17 min. Injection volume was 20 μl. The HPLC system was coupled to an Agilent G6495A Triple Quadrupole mass spectrometer equipped with an Agilent Jet Stream electrospray ionization source. Agilent MassHunter Acquisition software was used for data acquisition, and Agilent MassHunter Workstation software was used for data analysis.

### Statistical analysis

Statistical analysis and graphs preparation were performed using Prism GraphPad Software. T-test was used to analyze the significance of differences between various samples. Statistical significance was accepted if p < 0.05.

All experiments were performed in accordance with relevant guidelines and regulations.

## Results and discussion

### Yields of tobacco-derived extracellular vesicles from three various sources

**Fig. 1:**
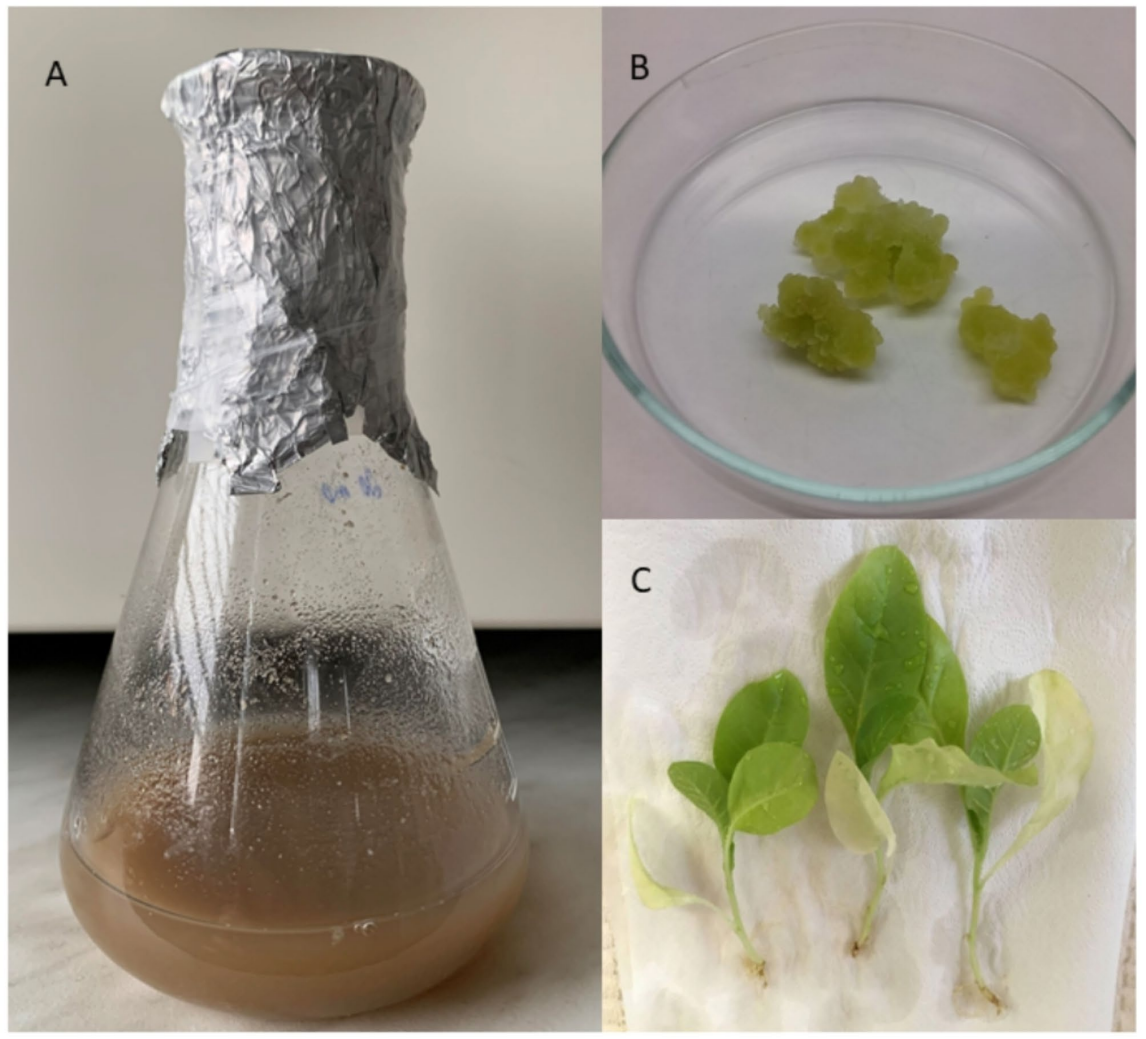
Tobacco-derived material used for isolation of extracellular vesicles. (**A**) suspension culture, (**B**) calli and (**C**) tobacco plants as plant material to obtain apoplastic fluid.

**Fig. 2:**
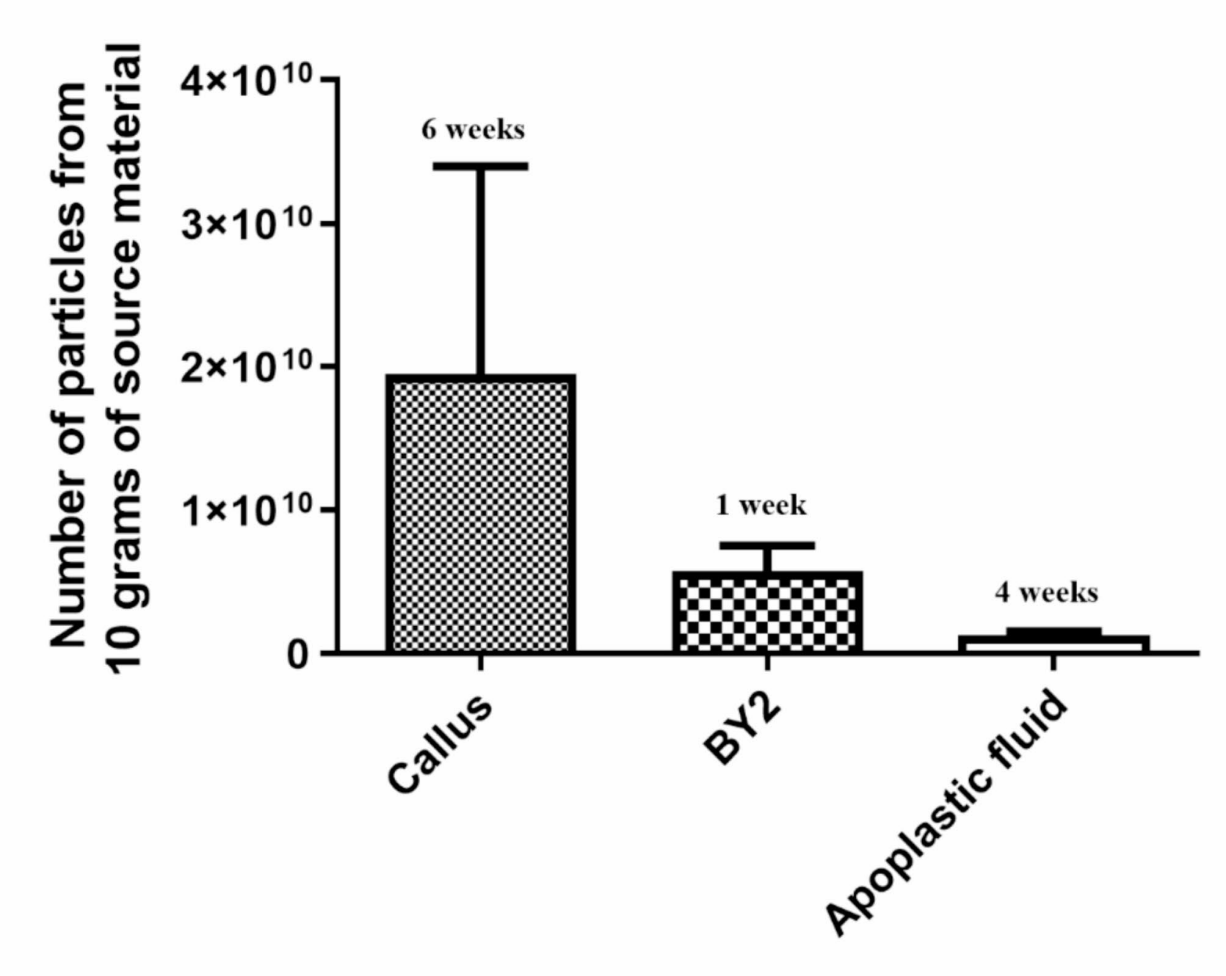
Yields of extracellular vesicles from different source materials, indicating the required cultivation time.

The production of extracellular vesicles can fluctuate based on both the isolation technique and their origin^27,41,42^. Three different tobacco sources were compared (Fig. 1), showing interesting biotechnological differences in yields. Calli and suspension cultures are mainly interesting because of the potential for sterile isolation, the convenient access to abundant material, and, in the case of suspension cultures, fast multiplication of material, compared to growing whole plants. Figure 2 illustrates the yields of pEVs as a number of particles isolated from 10 g of source material. Apoplastic fluid yielded the lowest amounts (1.24 × 10^9^ ± 5.28 × 10^8^) of extracellular vesicles, while callus cultures produced the highest yields (1.95 × 10^10^ ± 2.51 × 10^10^). Intermediate yields (5.70 × 10^9^ ± 3.12 × 10^9^) were observed for pEVs isolated from suspension cultures. Although suspension cell cultures originate from calli, the vesicle yields differ between the two. In mammalian systems, it has been shown that cells grown in 3D cultures produce more exosomes with different cargo molecules compared to those grown in traditional 2D cultures. This difference is attributed to the ability of 3D models to better replicate the natural cellular environment^43,44^. We hypothesize that the structural form of plant cell material similarly influences exosome production. In callus cultures, where cells are in close contact forming a compact mass, there may be enhanced cell-to-cell communication leading to increased exosome production. In contrast, suspension cultures lack this close cellular arrangement, potentially resulting in different exosome production dynamics.

In comparison to our study, different results between yields of pEVs isolated from *Arabidopsis thaliana* callus and apoplastic fluid were previously demonstrated^45^. In the study the vesicle concentration yields were determined relative to 1 g of callus or plant biomass of *A. thaliana*. The measured concentration from callus was 1.8 × 10^10^ particles/g and from apoplastic fluid 2.9 × 10^10^ particles/g. As already discussed by Yugay et al.^45^, the secretion of pEVs is influenced by physiological state of the plant and its cells during the harvest period, highlighting the dynamic nature of pEVs biogenesis in response to cellular growth. They assume the lower levels of pEVs in callus cultures can be attributed to its dedifferentiated state with no required heightened levels of active communication^45^. However, in study focusing on metabolic profiles of callus generated from tomato cotyledons and shoot regenerated from callus, the result show that the transformation of cotyledon to callus involves major shifts in metabolic profiles, increasing some metabolic pathways^46^. The changes in metabolic pathways of callus may lead to changes in production of pEVs, however more detailed research is needed in this area.

While considering the differences in yields is important, it is also crucial to account for the time required for growth or cultivation to obtain the specified input amount, especially if pEVs are to be used for biotechnological purposes. For the plant material tested, the cultivation times vary significantly: it takes four weeks to grow tobacco plants suitable for isolating apoplastic fluid, six weeks to generate the required quantity of callus, and only one week to achieve the necessary input amount from suspension culture. Despite the observed highest yield of pEVs from 10 g of callus, the practicality leans towards suspension cultures due to the challenges in growing a large amount of callus. The need for space for large amount of petri dishes poses a limitation, often restricting the number of petri dishes that can be inoculated simultaneously. As a result, suspension cultures prove more efficient in terms of space utilization and, consequently, yield higher quantities of pEVs within a shorter timeframe. Moreover, options such as increasing media volume in the flask, utilizing larger flasks, or using high-volume bioreactors provide practical solutions for enhancing the yield of suspension cultures. These alternatives offer the flexibility to upscale production significantly, allowing the cultivation of several liters of suspension cultures. This scalability factor makes suspension cultures more adaptable to larger-scale production and facilitates the optimization of pEVs yield in a more efficient and resource-effective manner which might be important mainly in case of selecting plant material for pEVs production for biotechnological purposes.

The lack of comparative studies focusing on yields of plant extracellular vesicles indeed highlights a potential gap in the current research landscape. Yield is a critical parameter that can significantly impact the feasibility and scalability of processes related to pEVs isolation and utilization.

### The efficiency of loading of tobacco-derived extracellular vesicles

**Fig. 3:**
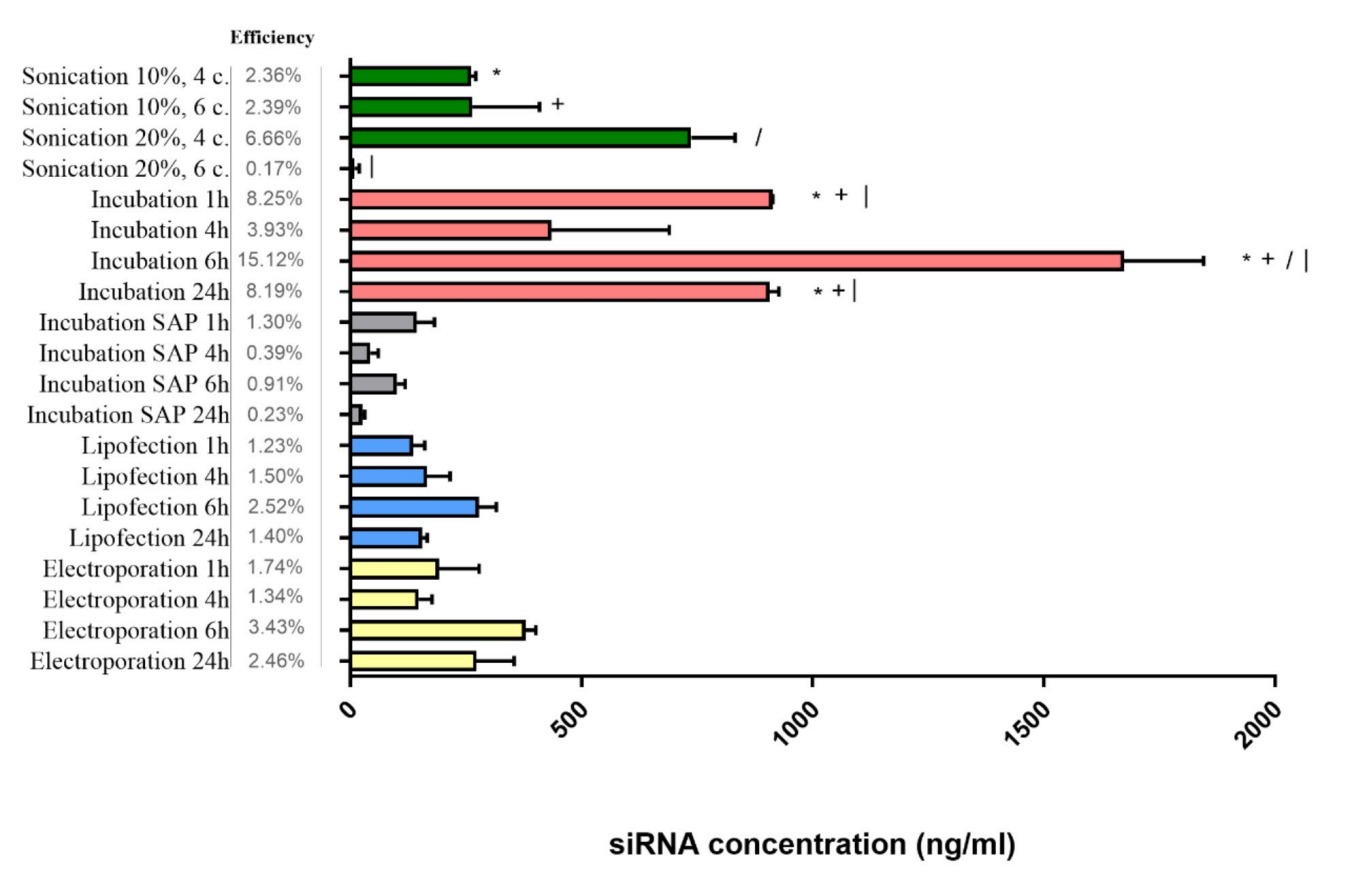
Concentration of siRNA loaded into callus-derived extracellular vesicles, showing the efficiency of the different loading methods. Significant differences (p < 0.05) between sonication and incubation variations are indicated by symbols next to each bar.

Figure 3 illustrates the concentration of siRNA (ng/ml) loaded into calli-derived pEVs using diverse methods and their corresponding efficiency. Significant differences were observed within certain loading groups (sonication, incubation etc.). The lowest efficiency (0.2%) was noted when using sonication with 20% amplitude and 6 cycles, and this efficiency was significantly different from those observed with sonication with 10% or 20% amplitude in 4 cycles. Within the incubation group, a significant difference was also identified, particularly in achieving the highest loading efficiency of all (15%) of siRNA into callus-derived pEVs, which was accomplished through a 6-h incubation period. No significant differences were noted in the efficiency among variations of incubation supplemented with saponin, as well as lipofection and electroporation, except for electroporation followed by 4 and 6 h of incubation, where a significant difference was observed. To ensure clarity, Fig. 3 shows the significant differences among individual variations of sonication and incubation (selected and compared because of the highest efficiencies) as the loading types using symbols positioned beside the bar corresponding to each variation.

In summary, our observations indicate that the most efficient approach for loading siRNA into tobacco callus-derived pEVs is through incubation, particularly with a 6-h duration yielding the highest efficiency. Slightly lower efficiency was attained through sonication, employing 20% amplitude and four cycles of repetition.

**Fig. 4:**
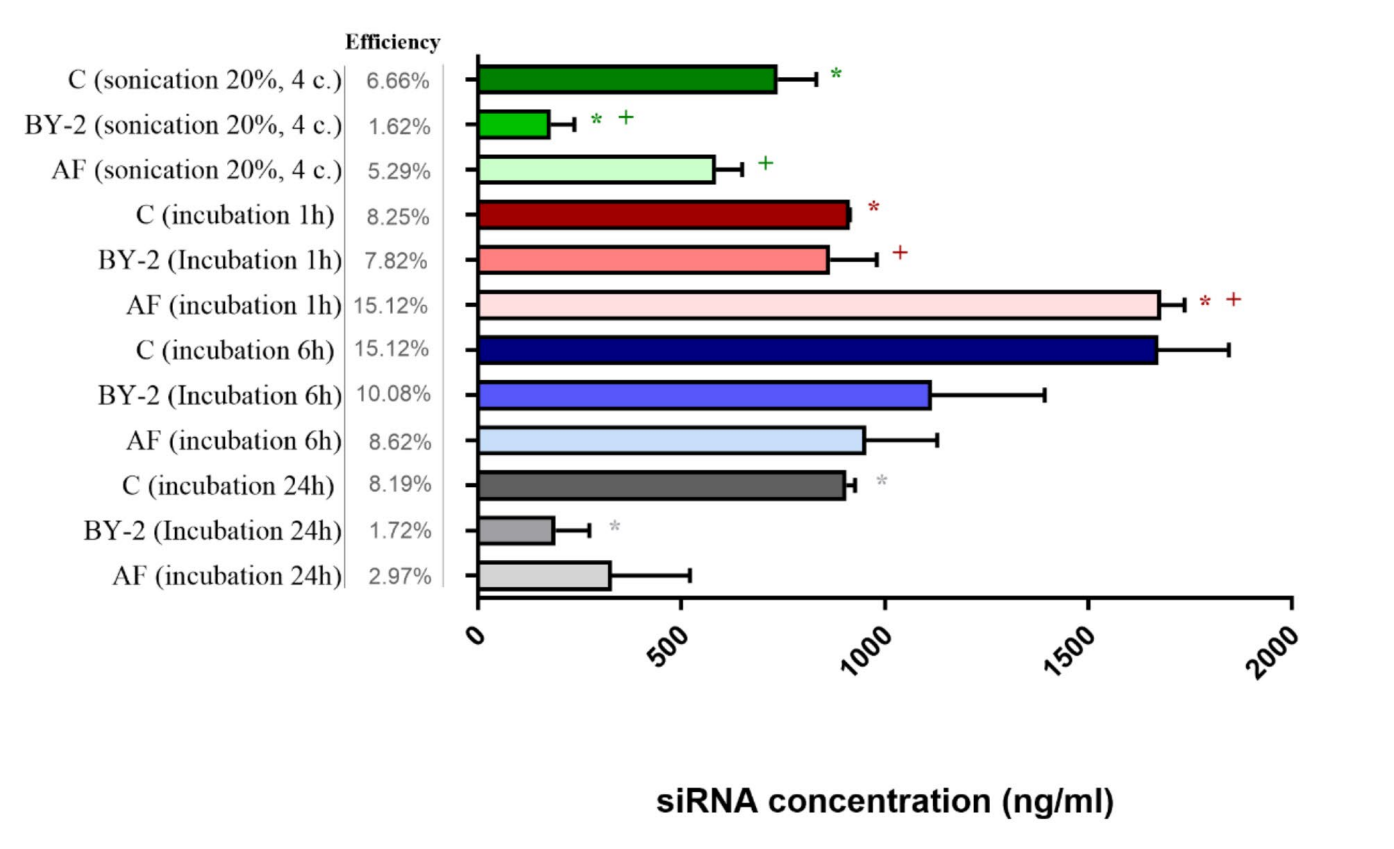
Concentration of siRNA loaded into extracellular vesicles from callus (C), suspension cultures (BY-2) and apoplastic fluid (AF). Loading efficiencies of different methods (sonication, incubation) are shown, with significant differences (p < 0.05) indicated by symbol next to each bar.

In our subsequent experiments, the most effective variations in cy5-siRNA loading efficiency was investigated among different methods applied to pEVs isolated from various tobacco sources, including apoplastic fluid, calli, and suspension cultures. Various loading methods were selected based on our previous results to assess their effectiveness. Including incubation (1, 6 and 24 h), as well as sonication with 20% amplitude and four cycles of repetition for our experimental variations. Significant differences (shown in Fig. 4) were observed within individual loading groups. In comparing the increasing efficiency, a consistent pattern in pEVs from both calli and suspension cultures was observed, where the loading method efficiency follows the order (from lowest to highest): sonication 20% amplitude, 4 cycles; incubation for 24 h; 1-h incubation, and 6-h incubation. For vesicles derived from apoplastic fluid, the loading efficiency exhibits a slightly different trend, increasing in the following order: 24-h incubation, sonication 20%, 4 cycles; incubation for 6 h, and 1-h incubation.

In conclusion, the most efficient method for loading siRNA into plant vesicles derived from callus, suspension culture, and apoplastic fluid was shown to be incubation. The optimal incubation time may vary based on the plant material used for isolating plant EVs. For tobacco vesicles, either a 1-h or 6-h incubation proves effective. Additionally, it was shown that in some cases, the loading efficiency was significantly higher when using pEVs from callus and/or apoplastic fluid. In contrast, there was not such a trend observed when utilizing pEVs from suspension culture. These results highlight the importance of optimizing EV loading procedures, as loading efficiency can vary not only based on the chosen method of loading but also on the type of vesicles used. The procedure for loading pEVs with siRNA is not well characterized, however, there are already some publications that primarily focus on loading mammalian extracellular vesicles. Number of methods were previously tested for EVs loading and several of them were shown to be effective. However, some limitations, such as the loading efficiency or damage caused during loading procedure, can affect the final application. Most results vary depending on type of loaded vesicles, loading method and experimental setups^47^. El-Andaloussi et al.^48^ reached 25% siRNA loading efficiency using electroporation under specific conditions: 400 µl of a sample consisting of an electroporation mixture prepared by mixing exosomes derived from dendritic cells and siRNA in a 1:1 (wt/wt) ratio in electroporation buffer, with the final concentration of exosomes in the mixture not exceeding 0.5 µg/µl^48^. Although these results show a high loading efficiency using electroporation, it has previously been reported as an inefficient method for loading siRNA into EVs due to the potential for siRNA aggregation, which can lead to misinterpretation of the loading efficiency results^49^. It was presented in another study that electroporation loading efficiency is influenced not only by exosome concentration, but also by electroporation intensity. The concentration of exosomes determined as the most effective was between 0.25 and 1 µg/µl with decrease of efficiency when using higher exosome concentration. Examination of six different voltage settings revealed that voltages between 150 and 250 V were most effective for loading siRNA. The electroporation efficiency was determined using FACS for the detection of fluorescently labeled siRNA in EVs. The percentage of plasma exosomes containing fluorescent signal reached 35%. However, they noted that the optimal electroporation settings vary for different types of exosomes^50^. Sonication was also tested for delivery of siRNA into animal EVs. It was reported that the efficiency of siRNA loading into HEK297T-derived EVs was 3.2% in comparison to 1% efficiency when sonication was not used. The experiments involved incubation of 1000 pmol siRNA with 100 µg of EVs for 30 min, followed by sonication in water bath sonicator. Same group also showed, using sonication for siRNA loading does not lead to siRNA aggregation as it was previously described for electroporation^51^. Moreover, vesicles derived from mesenchymal stem cells were loaded with siRNA using basic incubation for 2–3 h at 37 °C. The final loading efficiency was established using NanoSight. The concentration ratio of the fluorescent vs nonfluorescent exosomes was defined as the loading efficiency and it reached 33.6%^52^. Overall, the choice of siRNA loading method significantly impacts the efficiency and functionality of the resultant EVs. These findings suggest that method-specific optimizations are crucial for maximizing the therapeutic potential of siRNA-loaded EVs. Future research should continue to explore and refine these loading techniques to enhance their application in gene-silencing therapies not only in animal EVs, but also in pEVs.

**Fig. 5:**
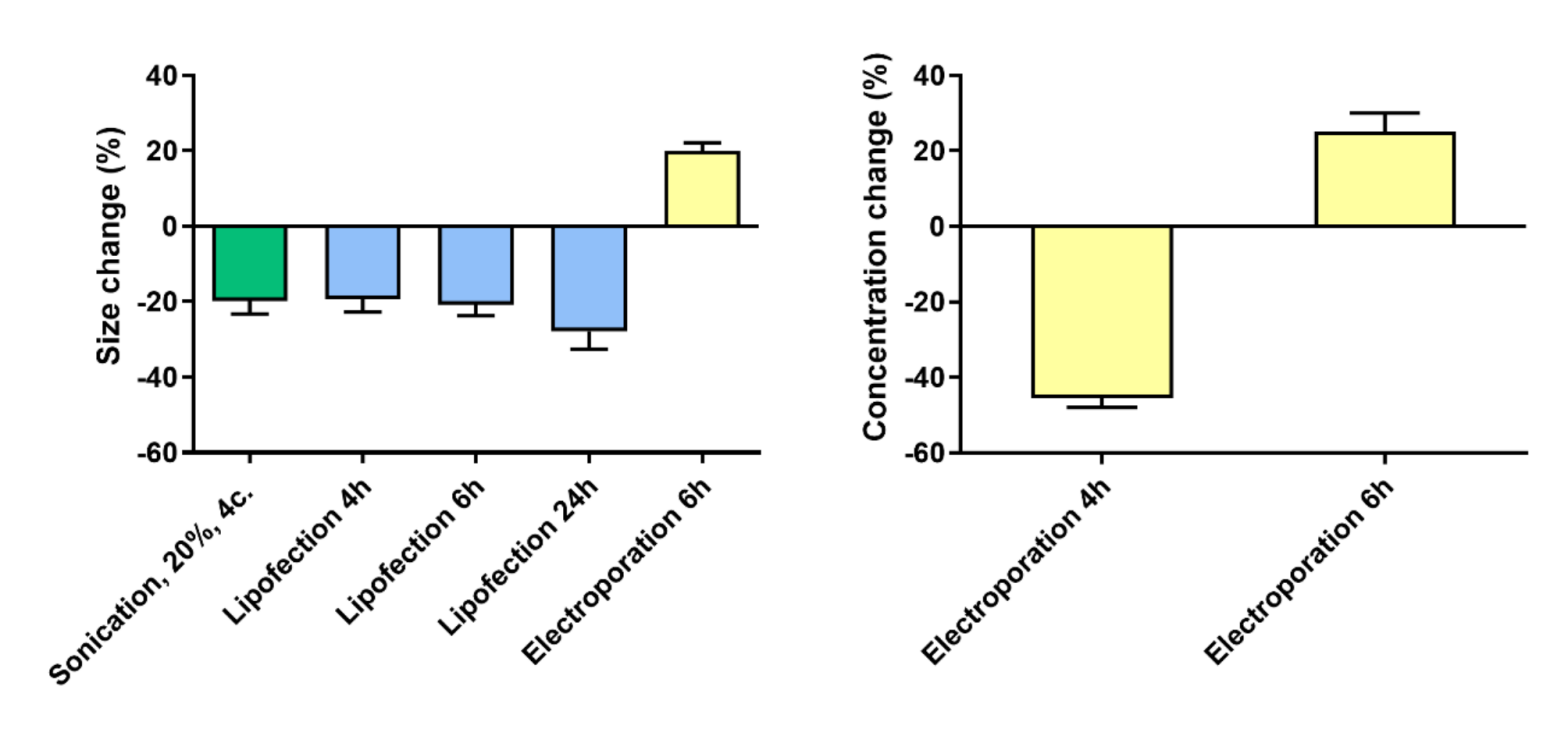
Effect of loading methods on vesicle size (left) and concentration (right). Significant changes (%) in both factors are shown comparing pre- and post-loading conditions.

To gain fundamental insights into the impact of loading methods on plant-derived vesicles, vesicle size and concentration both before and after the introduction of siRNA were measured. Figure 5 shows the effect of loading methods on vesicle size and concentration, revealing significant changes in both factors pre- and post-loading. Using other methods did not lead to significant differences, therefore they were not included in the figure. Instances of size reduction or size increase were observed, possibly attributed to the loading method inducing breakage in tobacco vesicles, subsequently leading to the formation of new vesicles with varying sizes, highlighting the dynamic nature of vesicles under diverse loading conditions.

Significant differences in size after cy-5 siRNA loading into pEVs were observed in five cases. When sonication with 20% amplitude and 4 cycle repetition was used, significant decrease (-19.8%) was observed, however the highest fluorescent signal of cy5-siRNA was observed using this variant. We suggest that the change in size may be attributed to the damage inflicted on the membrane of pEVs by ultrasonic waves and subsequent reassembly of vesicles. Sonication might also eliminate the presence of pEVs aggregates, as low-power sonication is commonly used to eliminate EVs aggregates after isolation, leading to difference in size distribution^53^. Moreover, three variations of lipofection (4-, 6- and 24-h incubation after lipofection) led to significant decrease in vesicles size. The loading efficiency of the method remains the second lowest and the damage of extracellular vesicles might be significant. Our findings align with the well-known cytotoxic nature of lipofectamine, attributed to its strong cationic properties, that may lead to disruption of vesicle membranes, while changing their size^54^. The significant increase in pEVs size was observe only using electroporation followed by 6-h incubation. Also, the only significant changes in concentration sizes, where observed after using electroporation followed by 4-h incubation and electroporation followed by 6-h incubation. Electroporation involves applying an electric field to the vesicle environment. As the electric field force gradually increases, the lipid bilayer rearranges to form hydrophilic channels, allowing small molecules to enter exosomes^55^. Upon cessation of the external electric field, lipid molecules return to their original stable structure. Electroporation can also help disaggregate pEVs, possibly explaining the observed changes in size and concentration. In summary, when evaluating the efficiency of loading and uniformity of vesicles in terms of size and concentration before and after loading, basic incubation emerges as the most effective method. These findings show the importance of method optimization. Similar results were observed by Lamichhane et al.^51^ with no differences observed in size and concentration changes pre- and after-loading of HEK293T-derived EVs using sonication. EVs were sonicated for varying times from 0–180 s in the presence or absence of siRNA and EV size and concentration was quantified via NTA^51^.

The goal of the experiments was to comprehensively evaluate and compare the efficiency of loading methods in introducing cy5-siRNA into the tobacco-derived vesicles, as there isn’t an extensive body of research specifically focused on loading siRNA into plant extracellular vesicles. Each method represents a distinct approach, and our experimental design aimed to elucidate the most effective and reliable strategy for loading siRNA into these vesicles. By employing a range of loading techniques, insights into the nuances of each method’s performance, considering factors such as encapsulation efficiency and the potential impact on vesicle integrity were collected. These findings will contribute to a better understanding of the loading capabilities of tobacco derived vesicles, offering valuable information for future studies focused on utilizing these vesicles as RNA carriers as siRNA serves as a potent tool for gene expression inhibition at the post-transcriptional level, offering precise control in therapies. While nucleic acid drugs, including siRNA, possess high hydrophilicity and negative charges, their entry into cells is impeded by nucleases, leading to degradation. This instability has prompted the development of drug delivery systems for protection. Loading nucleic acid drugs, mainly into mammalian exosomes has emerged as a promising strategy, leveraging the superior characteristics of exosomes: low cytotoxicity, high cargo expression, and high stability. However, challenges persist in delivering negatively charged siRNA through cell membranes, and concerns exist about prolonged siRNA stability. Extracellular vesicles present a potential solution to these challenges, addressing inherent therapeutic issues associated with siRNA delivery^24,56^.

### The presence of secondary metabolites in isolated plant extracellular vesicles

**Fig. 6:**
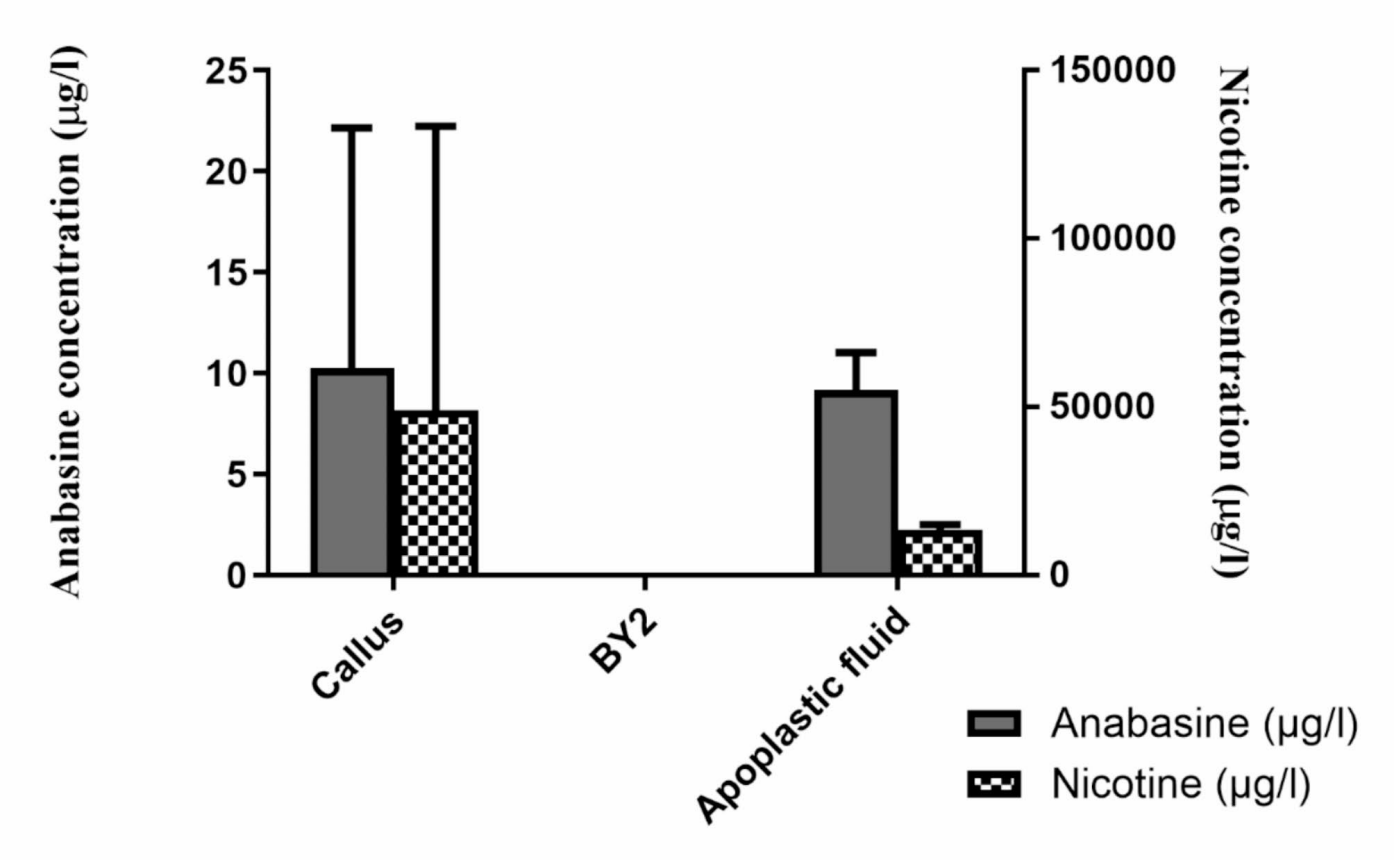
Nicotine and anabasine concentrations in tobacco extracellular vesicles derived from different sources.

In recent years, research has increasingly focused on identifying secondary metabolites within plant extracellular vesicles, anticipating potential benefits such as antibacterial, anti-inflammatory, anticancer, or antioxidant effects^12,57,58^. This is particularly relevant when using plant extracellular vesicles for medicinal purposes as a drug delivery tool. While existing publications have concentrated on secondary metabolites within pEVs isolated mainly from plant juices, limited knowledge exists regarding vesicles derived from calli or suspension cultures, as well as apoplastic fluid^25,26,57,59^. In our study, we aimed to investigate the presence of two secondary metabolites. The plant material was subjected to the same conditions of light and temperature, as described in the Material and Methods section. Vesicles were isolated from each type and conducted LC–MS/MS analysis to detect the presence of two tobacco-derived secondary metabolites, namely nicotine and anabasine. Our results revealed the presence of both anabasine and nicotine in callus-derived EVs (10.3 µg/l and 48,901 µg/l, respectively) and apoplastic fluid-derived EVs (9.2 µg/l and 13,563 µg/l, respectively), as shown in Fig. 6. However, vesicles originating from BY-2 suspension culture did not show detectable levels of the examined metabolites.

Our findings align with previously published results on alkaloid production in callus and BY-2 suspension cultures. Pinol et al.^60^ reported that alkaloid production in tobacco tissue cultures is influenced by the origin and age of the culture. They demonstrated that nicotine concentrations in callus cultures are slightly higher than in intact plants. Additionally, nicotine production is influenced by the concentration of auxin in the cultivation media, with 1.5 µM NAA identified as the optimal amount for nicotine production. It was also shown that alkaloid production in callus cultures increases after initiation, peaking around 6 or 7 weeks later and dropping after this period^60,61^. We confirmed this by analyzing pEVs from older callus cultures, where nicotine and anabasine were undetectable (data are not presented). In contrast, the BY-2 cell line, designed for rapid growth and multiplication, exhibits very low concentrations of alkaloid secondary metabolites, including nicotine and anabasine. However, it has been shown that alkaloid production in BY-2 cells can be significantly increased using elicitors, such as jasmonates, stress, or changes in media composition^62^. Although low metabolite levels in cells correspond to low packaging into pEVs, we chose to maintain the plant cultures in their natural state without applying stress. However, for biotechnological applications, the use of elicitors may be necessary to enhance secondary metabolite production in in vitro cultures. The presence of secondary metabolites within pEVs was previously confirmed in vesicles derived mainly from fruit or vegetable juices or from blended or dried plants^12,26,57^. We were interested if plant metabolites are present also in vesicles derived from explant cultures and apoplastic fluid. Although we confirmed the presence of nicotine and anabasine in callus and apoplastic fluid-derived pEVs, it is still uncertain what total composition of plant metabolites is packed into pEVs and how they are packed into vesicles during their biogenesis. Berger et al.^27^ investigated orange-derived pEVs and found that certain metabolites, including vitamin C and naringenin, were absent. This indicates that not all plant metabolites are necessarily encapsulated in pEVs^27^. There is limited number of studies focusing on metabolic analysis of pEVs and more research is needed in this area. Our study shows that extracellular vesicles derived not only from tobacco fresh plants but also from in vitro cultures can contain nicotine and anabasine, with their production dependent on the origin and the age of the culture. This finding is significant for future efforts in large-scale production of plant extracellular vesicles (pEVs), especially under sterile conditions where specific secondary metabolites may be required. We anticipate that in such scenarios, the use of elicitors to enhance metabolite production within plant cells will also result in their presence within extracellular vesicles.

## Conclusions

In the present study, a comparative analysis of plant extracellular vesicle yields derived from tobacco apoplastic fluid, calli, and suspension cultures (BY-2) was conducted. Our findings revealed variability in pEV yields depending on the source material from the same plant. While the highest yields were obtained from tobacco calli, their cultivation time and complexity led us to identify suspension cultures as the most effective for higher-volume pEV production. Although their yields are slightly lower, the ease with which low-volume cultivation in suspension cultures can be scaled up, with a weekly source material for vesicle isolation, makes them a more practical choice. Additionally, the efficiencies of small interfering RNA (siRNA) loading methods (sonication, incubation, incubation with saponin, lipofection, and electroporation) and assessed the size and concentration changes after loading were investigated. Our analysis on pEVs from calli, suspension cultures, and apoplastic fluid revealed a consistent trend for callus and suspension culture-derived EVs, where loading method efficiency followed the order: sonication (20% amplitude, 4 cycles), incubation for 24 h, 1-h incubation, and 6-h incubation. Interestingly, for vesicles derived from apoplastic fluid, the loading efficiency exhibited a slightly different trend, increasing in the following order: 24-h incubation, sonication (20%, 4 cycles), incubation for 6 h, and 1-h incubation. In conclusion, our results suggest that basic incubation of tobacco pEVs with siRNA, as well as sonication, emerged as the most effective methods for loading tobacco vesicles. Moreover, the presence of nicotine and anabasine within vesicles derived from calli and apoplastic fluid was detected, highlighting that pEVs can contain secondary metabolites even when isolated from young in vitro cultures. This underscores the potential significance of these findings for future efforts in producing plant extracellular vesicles with therapeutic applications.

## Data Availability

The datasets generated during and/or analysed during the current study are available in the Zenodo repository, https://doi.org/10.5281/zenodo.13589863.

## Abbreviations

BAP: 6-Benzylaminopurine
EVs: Extracellular vesicles
MS: Murashige–Skoog medium
NAA: 1-Naphthaleneacetic acid
NTA: Nanoparticle tracking analysis
pEVs: Plant extracellular vesicles
SAP: Saponin
2,4-D: 2,4-Dichlorophenoxyacetic acid
